# MGI short-read genome assemblies of *Pythium insidiosum* (reclassified as *Pythium periculosum*) strains Pi057C3 and Pi050C3

**DOI:** 10.1186/s13104-023-06587-6

**Published:** 2023-11-06

**Authors:** Theerapong Krajaejun, Preecha Patumcharoenpol, Thidarat Rujirawat, Weerayuth Kittichotirat, Sithichoke Tangphatsornruang

**Affiliations:** 1 Department of Pathology, Faculty of Medicine, Ramathibodi Hospital, Mahidol University, Bangkok, Thailand; 2 Interdisciplinary Graduate Program in Bioscience, Faculty of Science, Kasetsart University, Bangkok, Thailand; 3 Research Center, Faculty of Medicine, Ramathibodi Hospital, Mahidol University, Bangkok, Thailand; 4 Systems Biology and Bioinformatics Research Group, Pilot Plant Development and Training Institute, King Mongkut’s University of Technology Thonburi, Bangkhuntien, Bangkok, Thailand; 5 National Omics Center, National Science and Technology Development Agency, Pathum Thani, 12120 Thailand

**Keywords:** *Pythium insidiosum*, Pythiosis, Genome, MGI, Next-generation sequencing

## Abstract

**Objectives:**

*Pythium insidiosum* causes a difficult-to-treat infectious condition called pythiosis, with high morbidity and mortality. So far, genome data of at least 10 strains of *P. insidiosum*, primarily classified in the phylogenetic clades I and II, have been sequenced using various next-generation sequencing platforms. The MGI short-read platform was employed to obtain genome data of 2 clade-III strains of *P. insidiosum* (recently reclassified as *Pythium periculosum*) from patients in Thailand and the United States. This work is a part of our attempt to generate a comprehensive genome database from diverse pathogen strains.

**Data description:**

A 150-bp paired-end library was prepared from a gDNA sample of *P. insidiosum* (*P. periculosum*) strains Pi057C3 and Pi050C3 (also known as ATCC90586) to generate draft genome sequences using an MGISEQ-2000RS sequencer. As a result, for the strain Pi057C3, we obtained a 42.5-Mb assembled genome (164x coverage) comprising 14,134 contigs, L50 of 241, N50 of 45,748, 57.6% CG content, and 12,147 ORFs. For the strain Pi050C3, we received a 43.3-Mb draft genome (230x coverage) containing 14,511 contigs, L50 of 245, N50 of 45,208, 57.7% CG content, and 12,249 ORFs. The genome sequences have been deposited in the NCBI/DDBJ databases under the accession numbers JAKCXM000000000.1 (strain Pi057C3) and JAKCXL000000000.1 (strain Pi050C3).

## Objective

*Pythium insidiosum* is a devastating oomycete pathogen that causes a difficult-to-treat infectious condition called pythiosis, which comes with high morbidity and mortality [1–4]. The pathogen has been taxonomized in the Phylum Oomycota and Clades Stramenopiles of Superkingdom Eukaryota (https://www.ncbi.nlm.nih.gov/taxonomy). Evolution might play an important role in differentiating *P. insidiosum* from the other pathogenic oomycetes, which mostly infect plants, to become a highly virulent human/animal pathogen. Typing the ribosomal deoxyribonucleic acid (rDNA) sequence classifies *P. insidiosum* into three major clades, which are associated with its geographic distributions: clade-I strains from the Americas, clade-II strains from the Americas, Asia, and Australia, and clade-III strains mainly from Thailand and the United States [5, 6]. Miraglia et al. recently reclassified the *P. insidiosum* strains in the genotype clade-III as *Pythium periculosum* based on the population genetic analysis [7]. So far, the draft genome data of at least 10 strains of *P. insidiosum* have been sequenced using various next-generation sequencing (NGS) platforms (i.e., 454, Illumina, and PacBio) and deposited in the public databases [8–15]. Most of these strains are genotypes clade-I and -II. The current study employed the MGI short-read NGS platform to obtain genome data of 2 additional strains, phylogenetically grouped in clade-III and isolated from human patients living in Thailand and the United States. This work is a part of our attempt to generate a comprehensive genome database from diverse strains of *P. insidiosum* for downstream comparative and population genomic analyses to explore microbial evolution and pathogenicity. The obtained genome information will serve our long-term goal of unveiling the pathogenesis mechanism and, ultimately, identifying the pathogen’s efficient diagnostic and therapeutic target.

## Data description

This study reports draft genome sequences of two phylogenetic clade-III strains of *P. insidiosum* (reclassified as *P. periculosum*). The first strain, Pi057C3, was isolated from a human pythiosis patient in Thailand. The second strain, Pi050C3 (also known as strain ATCC90586), was isolated from a human pythiosis patient in the United States. Species and genotype identifications of these organisms were achieved by rDNA sequence homology analysis [5, 6]. The organisms were incubated in Sabouraud dextrose broth and shaken at 37 °C for seven days. The organism was harvested for genomic DNA (gDNA) extraction using the optimized method developed for *P. insidiosum* [16]. Following the company’s instruction, the obtained gDNA (300 ng) from each strain was subjected to the library construction using an MGI Eazy FS Library Prep Kit (MGI Tech, Shenzhen, China). The 150-bp paired-end sequencing was conducted on an MGISEQ-2000RS sequencer to generate the shotgun genome sequences.

46,422,128 reads comprising 6,963,319,140 bases from strain Pi057C3 and 66,245,520 reads comprising 9,936,827,970 bases from strain Pi050C3 were obtained. All cleaned reads were de novo sequence assembled using SPAdes v3.14.0 [17] with its default settings, except the size of k-mers, which was adjusted to k21, k33, k55, k77, and k99. As a result, a draft assembled genome of strain Pi057C3 contained 14,134 contigs with an average length of 3,010 bases (range: 200–937,540), L50 of 241, N50 of 45,748, total bases of 42,546,069, CG content of 57.6%, and genome coverage of 164x, whereas that of strain Pi050C3 contained 14,511 contigs with an average length of 2,984 bases (range: 200–964,331), L50 of 245, N50 of 45,208, total bases of 43,294,389, CG content of 57.7%, and genome coverage of 230x. The draft genomes had completeness of 99% (for strain Pi057C3) and 96% (for strain Pi050C3), as indicated by the BUSCO 5.4.5 software, using a well-defined set of 100 highly-conserved genes from Stramenopiles [18]. Augustus v3.3.3 [19] assigned 12,147 open reading frames (ORFs) in the genome of strain Pi057C3 and 12,249 ORFs in the genome of strain Pi050C3. Protein sequences from both strains were compared to the NCBI protein sequence database using BLASTP software. Based on functional classification using Clusters of Orthologous Groups (COG) [20], 6.8%, 10.1%, 11.1%, and 72.0% of the genes from strain Pi057C3 and 6.6%, 9.9%, 11.0%, and 72.5% of the genes from strain Pi050C3 can be respectively assigned to the following categories: (i) information storage and processing, (ii) cellular processes and signaling, (iii) metabolism, and (iv) poorly characterized or hypothetical proteins. The assembled genome sequences have been stored in the National Center for Biotechnology Information (NCBI) and DNA Data Bank of Japan (DDBJ) databases and are publicly accessible via the accession numbers JAKCXM000000000.1 (strain Pi057C3) and JAKCXL000000000.1 (strain Pi050C3) (Table 1).

In summary, the genomes of *P. insidiosum* (*P. periculosum*) strains Pi057C3 (genome size: 42.5 Mb) and PI050C3 (genome size: 43.3 Mb) isolated from Thai and American patients with pythiosis have been sequenced and made publicly available. These genome data will be analyzed as a part of our population genomic study to elucidate the genome-scale biodiversity of this pathogen.

**Table 1:** Overview of data files/data sets

Label	Name of data file/data set	File types (file extension)	Data repository and identifier (DOI or accession number)
Data file 1	*Pythium insidiosum* strain Pi057C3, whole genome shotgun sequencing project	FASTA	GenBank (https://identifiers.org/ncbi/insdc:JAKCXM010000001.1 - https://identifiers.org/ncbi/insdc:JAKCXM010014134.1) [21]
Data file 2	*Pythium insidiosum* strain Pi050C3 (ATCC90586), whole genome shotgun sequencing project	FASTA	GenBank (https://identifiers.org/ncbi/insdc:JAKCXL010000001.1 - https://identifiers.org/ncbi/insdc:JAKCXL010014511.1) [22]

## Limitations

We used the MGISEQ-2000RS sequencer to generate short reads for the draft genome assembly of the organism strains Pi057C3 and Pi050C3. A few limitations of such an NGS platform were noted as follows. Like the sequencing-by-synthesis technique (SBS) used in other platforms (i.e., Illumina), the MGI SBS generates a sequence library employing DNA amplification, which, to some extent, results in sequence coverage biases and substitution errors. The draft genome of each strain was obtained from the construction of a 150-bp paired-end library, leading to relatively higher contig numbers (14,134 contigs for strain Pi057C3 and 14,511 contigs for strain Pi050C3) and relatively smaller genome sizes (42.5 Mb for strain Pi057C3 and 43.3 Mb for strain Pi050C3), when compared to the reference *P. insidiosum* genome, which was assembled from 4 paired-end and mate-pair libraries, resulting in fewer contigs (n = 1,192) and bigger genome size (53.2 Mb) [10].

## Data Availability

The draft genome sequence of the *P. insidiosum* strain Pi057C3 comprising 14,134 contigs (accession numbers JAKCXM010000001-JAKCXM010014134) is available in GenBank here: https://identifiers.org/ncbi/insdc:JAKCXM000000000.1 [21]. The draft genome sequence of the *P. insidiosum* strain Pi050C3 comprising 14,511 contigs (accession numbers JAKCXL010000001-JAKCXL010014511) is available in GenBank here: https://identifiers.org/ncbi/insdc:JAKCXL000000000.1 [22].

## Abbreviations

DDBJ: DNA Data Bank of Japan
gDNA: Genomic deoxyribonucleic acid
NCBI: National Center for Biotechnology Information
NGS: Next-generation sequencing
ORF: Open reading frame
rDNA: Ribosomal deoxyribonucleic acid

## References

[1] Yolanda H, Krajaejun T (2022). Global distribution and clinical features of pythiosis in humans and animals. J Fungi.

[2] Yolanda H, Krajaejun T (2021). History and perspective of Immunotherapy for Pythiosis. Vaccines.

[3] Krajaejun T, Sathapatayavongs B, Pracharktam R, Nitiyanant P, Leelachaikul P, Wanachiwanawin W (2006). Clinical and epidemiological analyses of human pythiosis in Thailand. Clin Infect Dis.

[4] Gaastra W, Lipman LJ, De Cock AW, Exel TK, Pegge RB, Scheurwater J (2010). Pythium insidiosum: an overview. Vet Microbiol.

[5] Rujirawat T, Sridapan T, Lohnoo T, Yingyong W, Kumsang Y, Sae-Chew P (2017). Single nucleotide polymorphism-based multiplex PCR for identification and genotyping of the oomycete Pythium insidiosum from humans, animals and the environment. Infect Genet Evol.

[6] Schurko AM, Mendoza L, Lévesque CA, Désaulniers NL, de Cock AWAM, Klassen GR (2003). A molecular phylogeny of Pythium insidiosum. Mycol Res.

[7] Miraglia BM, Mendoza L, Rammohan R, Vilela L, Vilela C, Vilela G (2022). Pythium insidiosum complex hides a cryptic novel species: Pythium Periculosum. Fungal Biol.

[8] Kittichotirat W, Patumcharoenpol P, Rujirawat T, Lohnoo T, Yingyong W, Krajaejun T (2017). Draft genome and sequence variant data of the oomycete Pythium insidiosum strain Pi45 from the phylogenetically-distinct Clade-III. Data Brief.

[9] Patumcharoenpol P, Rujirawat T, Lohnoo T, Yingyong W, Vanittanakom N, Kittichotirat W (2018). Draft genome sequences of the oomycete Pythium insidiosum strain CBS 573.85 from a horse with pythiosis and strain CR02 from the environment. Data Brief.

[10] Rujirawat T, Patumcharoenpol P, Lohnoo T, Yingyong W, Lerksuthirat T, Tangphatsornruang S (2015). Draft genome sequence of the pathogenic oomycete Pythium insidiosum strain Pi-S, isolated from a patient with Pythiosis. Genome Announc.

[11] Ascunce MS, Huguet-Tapia JC, Braun EL, Ortiz-Urquiza A, Keyhani NO, Goss EM (2016). Whole genome sequence of the emerging oomycete pathogen Pythium insidiosum strain CDC-B5653 isolated from an infected human in the USA. Genomics Data.

[12] Krajaejun T, Kittichotirat W, Patumcharoenpol P, Rujirawat T, Lohnoo T, Yingyong W (2018). Data on whole genome sequencing of the oomycete Pythium insidiosum strain CBS 101555 from a horse with pythiosis in Brazil. BMC Res Notes.

[13] Rujirawat T, Patumcharoenpol P, Lohnoo T, Yingyong W, Kumsang Y, Payattikul P (2018). Probing the Phylogenomics and putative pathogenicity genes of Pythium insidiosum by Oomycete Genome analyses. Sci Rep.

[14] Krajaejun T, Kittichotirat W, Patumcharoenpol P, Rujirawat T, Lohnoo T, Yingyong W (2020). Draft genome sequence of the oomycete Pythium destruens strain ATCC 64221 from a horse with pythiosis in Australia. BMC Res Notes.

[15] Krajaejun T, Kittichotirat W, Patumcharoenpol P, Rujirawat T, Lohnoo T, Yingyong W (2021). Genome data of four Pythium insidiosum strains from the phylogenetically-distinct clades I, II, and III. BMC Res Notes.

[16] Lohnoo T, Jongruja N, Rujirawat T, Yingyon W, Lerksuthirat T, Nampoon U (2014). Efficiency comparison of three methods for extracting genomic DNA of the pathogenic oomycete Pythium insidiosum. J Med Assoc Thai.

[17] Bankevich A, Nurk S, Antipov D, Gurevich AA, Dvorkin M, Kulikov AS (2012). SPAdes: a new genome assembly algorithm and its applications to single-cell sequencing. J Comput Biol J Comput Mol Cell Biol.

[18] Manni M, Berkeley MR, Seppey M, Simão FA, Zdobnov EM (2021). BUSCO Update: Novel and Streamlined Workflows along with broader and deeper phylogenetic Coverage for Scoring of Eukaryotic, Prokaryotic, and viral genomes. Mol Biol Evol.

[19] Stanke M, Keller O, Gunduz I, Hayes A, Waack S, Morgenstern B. AUGUSTUS: ab initio prediction of alternative transcripts. Nucleic Acids Res. 2006;34 Web Server issue:W435–9. 10.1093/nar/gkl200 PMC1538822 16845043

[20] Galperin MY, Makarova KS, Wolf YI, Koonin EV (2015). Expanded microbial genome coverage and improved protein family annotation in the COG database. Nucleic Acids Res.

[21] Rujirawat T, Patumcharoenpol P, Kittichotirat W, Krajaejun T. *Pythium insidiosum* strain Pi057C3, whole genome shotgun sequencing project. GenBank. 2022. https://identifiers.org/ncbi/insdc:JAKCXM000000000.1.

[22] Rujirawat T, Patumcharoenpol P, Kittichotirat W, Krajaejun T. *Pythium insidiosum* strain Pi050C3, whole genome shotgun sequencing project. GenBank. 2022. https://identifiers.org/ncbi/insdc:JAKCXL000000000.1.

